# Calibration of Action Cameras for Photogrammetric Purposes

**DOI:** 10.3390/s140917471

**Published:** 2014-09-18

**Authors:** Caterina Balletti, Francesco Guerra, Vassilios Tsioukas, Paolo Vernier

**Affiliations:** 1 IUAV di Venezia-S. Croce 191, 30135 Venezia, Italy; E-Mails: balletti@iuav.it (C.B.); guerra2@iuav.it (F.G.); vernier@iuav.it (P.V.); 2 Aristotle University of Thessaloniki, Univ. Box. 432, Thessaloniki 54124, Greece

**Keywords:** photogrammetry, calibration, undistortion, video

## Abstract

The use of action cameras for photogrammetry purposes is not widespread due to the fact that until recently the images provided by the sensors, using either still or video capture mode, were not big enough to perform and provide the appropriate analysis with the necessary photogrammetric accuracy. However, several manufacturers have recently produced and released new lightweight devices which are: (a) easy to handle, (b) capable of performing under extreme conditions and more importantly (c) able to provide both still images and video sequences of high resolution. In order to be able to use the sensor of action cameras we must apply a careful and reliable self-calibration prior to the use of any photogrammetric procedure, a relatively difficult scenario because of the short focal length of the camera and its wide angle lens that is used to obtain the maximum possible resolution of images. Special software, using functions of the OpenCV library, has been created to perform both the calibration and the production of undistorted scenes for each one of the still and video image capturing mode of a novel action camera, the GoPro Hero 3 camera that can provide still images up to 12 Mp and video up 8 Mp resolution.

## 1. Introduction

During the last few years, passive sensors providing visual image information [1], as well as the development of software solutions for the extraction of point clouds from a set of un-oriented images have received increasing attention, not only from the surveying community, but also from non-experts (such as archeologists). The reason is due to some clear advantages provided from these systems: the improved resolution and sensitivity of photo sensors and the availability of low cost off-the-shelf digital cameras which can be used in several photogrammetric applications, such as aerial mapping (e.g., aimed to structural and archaeological surveying) or emergency documentation.

Normally in photogrammetric applications, amateur cameras, semi-professional and professional cameras (dSLR) are not appropriate for measurement purposes. Making it necessary to calibrate them by estimating their intrinsic parameters in order to be able to extract accurate 3D metric information from their images [2]. The estimation of these geometric characteristics, *i.e.*, the focal length *(f)* of the lens, the coordinates of the centre of projection of the image (*x*_p_*, y*_p_), the radial lens distortion coefficients (*k*_1_, *k*_2_, *k*_3_) [2], is performed through the camera calibration process.

A full review of camera calibration methods and models developed in the last few years can be found in [3–5] describing the commonly adopted methods such as Tsai [6] or Zhang [7]. These are all based on the pinhole camera model including terms for modeling radial distortion. Nowadays there are several software applications (*i.e.*, Photomodeler, Agisoft Lens, iWitness, MicMac, 3DF Zephir, *etc.*), mainly produced by the computer vision scientific community, that can automatically perform camera self-calibration. They also offer the possibility to work with several cameras and sensors to obtain dense point clouds or 3D models suitable for different fields of applicFation.

In the wide panorama of low-priced consumer grade devices [8] (including nowadays even smartphones or other similar mobile devices) [9,10], action cameras are more widespread and have been used more and more during extreme activities such as free fall, parapent flying, underwater swimming and diving. Their photogrammetric use though has not been easy since they could not supply high resolution still images and video and additionally their geometry are far away from the theoretical model of central projection due to their wide angle or fish eye lenses.

Several approaches have recently been testing the use of the GoPro Hero 3 camera (GoPro, Inc., San Mateo, CA, USA) [11–13], particularly for the recording and/or photogrammetric use in underwater conditions even using a stereoscopic configuration to obtain better results or to produce models from immersive videos. However, this was not possible since the baseline of the cameras was very short [14–16].

The camera's characteristics are ideal not only for underwater conditions, but also in UAV configurations. The camera is very light and additionally it can be controlled through a WiFi connection from a cell phone or tablet [17].

## 2. Experimental Section

### 2.1. Camera Specifications

The weight (Table 1) and dimensions (Table 2) of the camera are ideal for photogrammetric applications, especially when it is necessary to place it on an UAV or on top of a diver's mask.

The GoPro Hero 3 camera has a rather big sensor which is able to provide four different still image resolutions (Table 3) and nine different video resolutions (Table 4).

The modes of the camera used to capture still images or video sequences vary depending on the needs of the application and the user demands. The frame rate of the coarser video resolution (WVGA 848 × 480) is about 240 FPS, while the finer resolution (4K Cin 4096 × 2160) can provide a minimum of 12 FPS (Table 4).

Additionally, the image dimensions ratio also varies and can be 4:3, 16:9 or 17:9. Therefore, the sensor part used to acquire the still images or the video sequences is not always the same. For this reason separate self-calibration procedures have to be applied in order to calculate the camera calibration parameters. Using our software, we have calibrated the GoPro camera in several of its resolutions, however for photogrammetric purposes the most useful is the largest one, hence for the accuracy assessment of our calibration procedure (see Section 3) we have used only 12 Mpixels GoPro images.

### 2.2. Calibration Model and Undistorted Images

A modified mathematical of the Brown Calibration model [18] has been used to determine all the appropriate parameters that determine the lens deformation appearing on an image [19]. The specific model uses odd and even order polynomial coefficients to model the radial and the tangential distortion of the lenses (Equations (1–4)):

(1) $$dx_{\text{radial}}=x\cdot (1+k_{1}\cdot r^{2}+k_{3}\cdot r^{4}+k_{5}\cdot r^{5})$$

(2) $$dy_{\text{radial}}=y\cdot (1+k_{1}\cdot r^{2}+k_{3}\cdot r^{4}+k_{5}\cdot r^{5})$$

(3) $$dx_{\tan}=x+[2\cdot p_{1}\cdot x\cdot y+p_{2}\cdot (r^{2}+2\cdot x^{2})]$$

(4) $$dy_{\tan}=y+[p_{1}\cdot (r^{2}+2\cdot y^{2})+2\cdot p_{2}\cdot x\cdot y]$$

**Figure 1.**
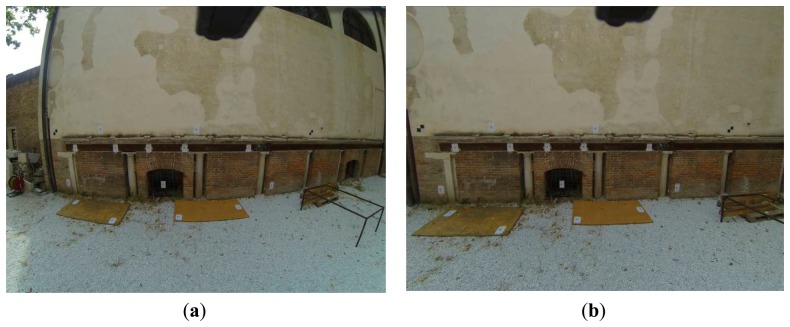
The (**a**) distorted image from the GoPro Hero 3 Camera and its (**b**) undistorted version. The undistorted image has been created using the special remapping function of OpenCV.

The developed software, containing all the procedures needed to calibrate not just the GoPro camera, but any imaging sensor that is able to create digital photographic images, is compiled in Microsoft Visual Studio 2010, using the functions of the graphics library OpenCV (version 2.4.3). Within the OpenCV library there are also functions that implement the modified Brown Calibration Model. With the use of certain image analysis and adjustment functions of OpenCV it was feasible to create a software application running in Windows OS that is able to:

- Identify a big number of ground control points (targets) on images printed on a special calibration sheet (chessboard or circle patterns);
- Calculate through adjustment all the camera calibration parameters; except for the radial and tangential calibration parameters the camera constant (*f*) value and the principal point's coordinates (*x_o_*, *y_o_*) are determined;
- And (optionally) create the undistorted (idealized) images (Figure 1).

The estimated accuracy of the calibration is given by the OpenCV routine that simultaneously calculates the extrinsic and intrinsic parameters of all the images used in the calibration process. The value is given in pixels and is referred to as Average Reprojection Error. It concerns the difference of target points location defined in the original images from their estimated location using the calculated exterior, interior and calibration parameters.

**Figure 2.**
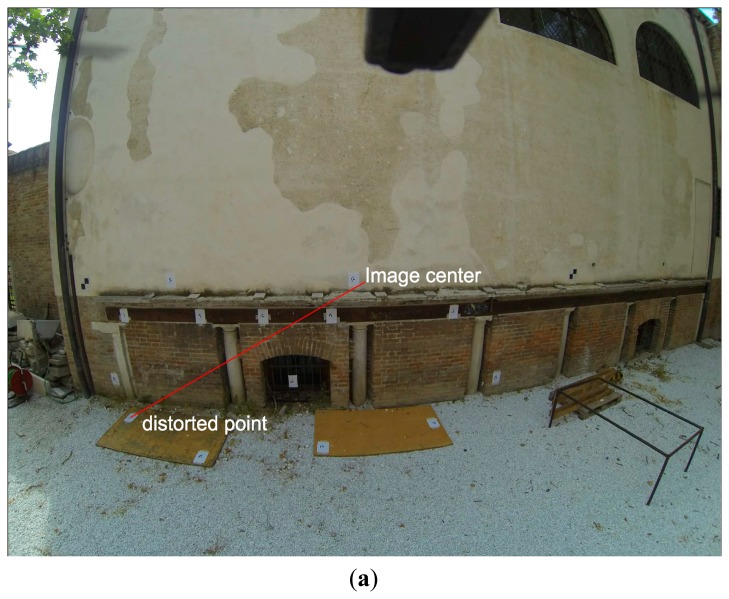

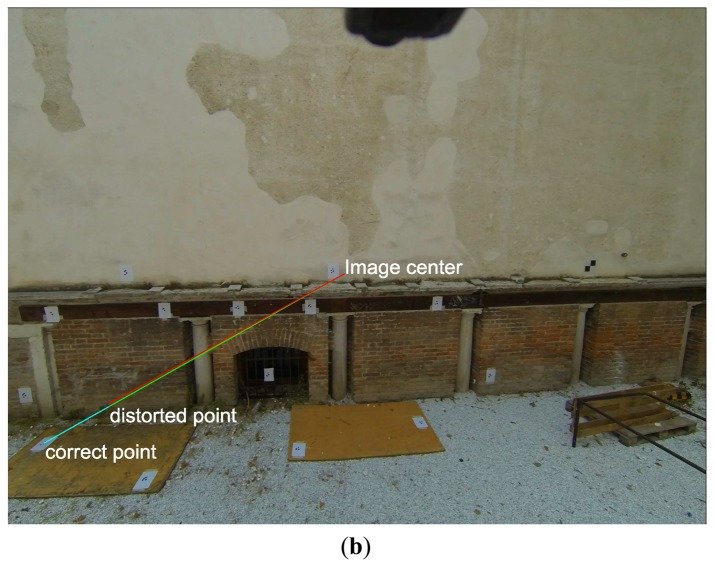
On the (**a**) original and (**b**) idealized images the deformation error is visualized.

**Figure 3.**
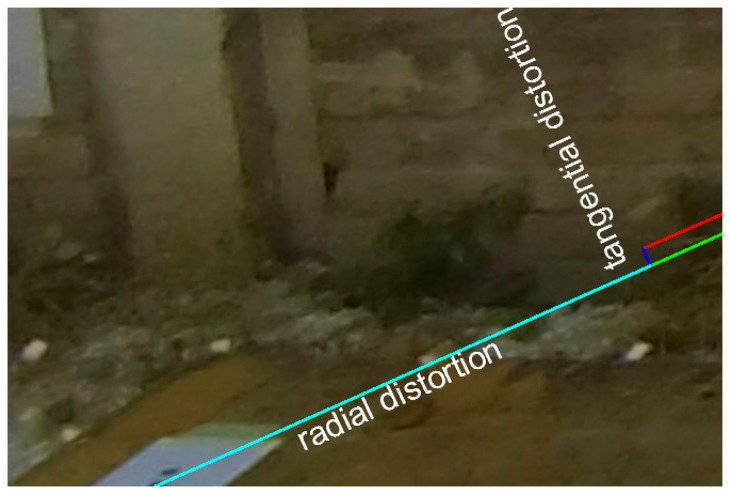
Detail of the distortions.

Examining the displacement of a point on the original (Figure 2a) and the idealized image (Figure 2b) we can identify the radial and tangential deformation. The radial deformation (cyan line) is collinear to the radius of the correct location of the point from the image center and the tangential (blue) line is vertical to the radius. Its tangential vector is relatively small to the total deformation error (Figure 3).

### 2.3. Recognized Problems and Finding Solutions

Although there exists an OpenCV sample software application (running under the Windows command line environment) able to deliver camera calibration, it cannot be used to calibrate high resolution digital cameras (more than 20 Mpixels). The specific software was originally created for the calibration of small frame cameras and especially for web cameras in Computer Vision Applications.

**Figure 4.**
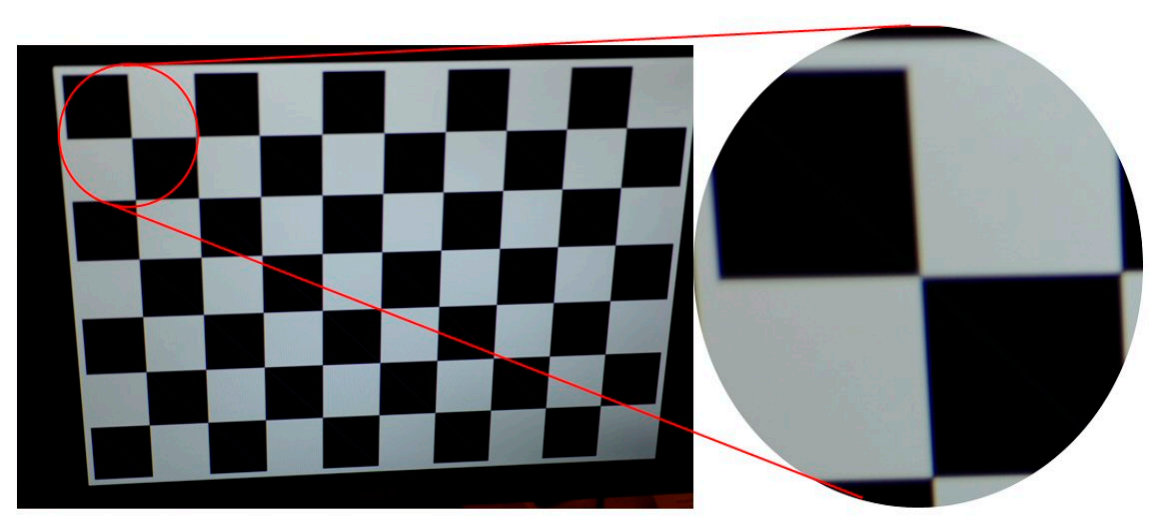
The upper left part of the image is appearing defocused. The software was unable to identify the intersection of the squares and perform the calibration.

Certain problems appeared when we tried to identify control points of the printed calibration sheet on high resolution images. Especially, when we tried to calibrate a full frame camera (Nikon D800 (NIKON CORPORATION, Tokyo, Japan)) of a 36.3 Mpixels resolution the software was unable to identify the points of a chessboard calibration field. The effect of defocus in the part of the image that was away from the center of the image (or the point in focus) introduced the biggest problem (Figure 4).

**Figure 5.**
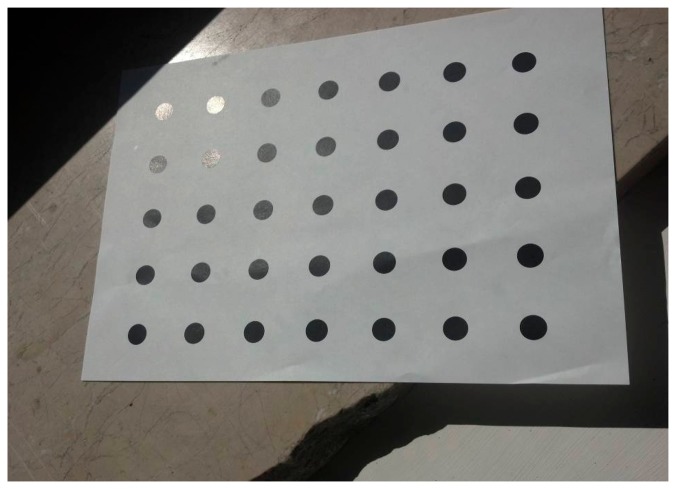
The gloss effect on the top left corner of the calibration field is giving brighter colors to some circles of the calibration field.

Therefore we decided to use a regular grid of circles as calibration field. In the case of the circular grid, the barycentric algorithm that we used to identify the exact location of the circles' centers, was not affected by the defocusing effect. Another issue appearing in some cases is the bad lighting of the calibration sheet during the image capture. Not all the people that want to calibrate their cameras have the experience and awareness to deliver appropriate calibration image shots, especially students that want to use their amateur cameras for photogrammetric recording purposes. Low luminance and a gloss effect (Figure 5) that might appear when the light is hitting the calibration field page from a certain angle might confuse the software and block the algorithm from identifying the control points on the page.

In general all calibration software functions as a “black box” application and the user has no option to adjust the settings of the algorithms to identify the control points.

**Figure 6.**
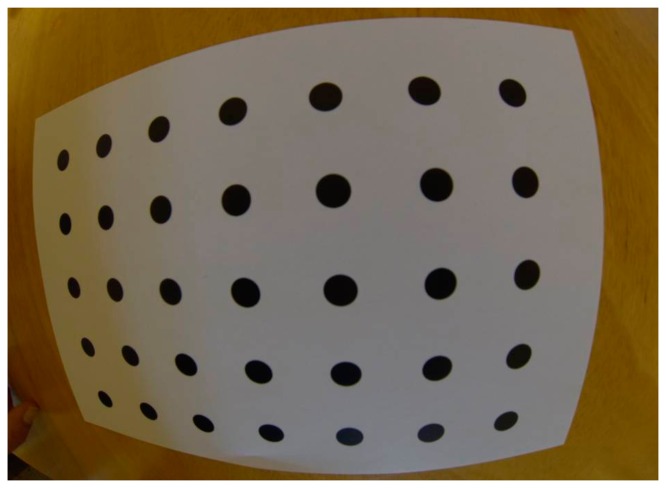
In case of wide angle lens cameras and when the inclination of the camera is big the circle's diameter of the control points might be three times smaller at the edges of the calibration image.

When using wide angle lenses and in cases where the camera location has a big inclination with the calibration page, the size of the control points' circles varies a lot. In some cases the circle radius of the most remote circles could be three times smaller than those close to the lens, especially in the case of wide angle lenses (Figure 6).

**Figure 7.**
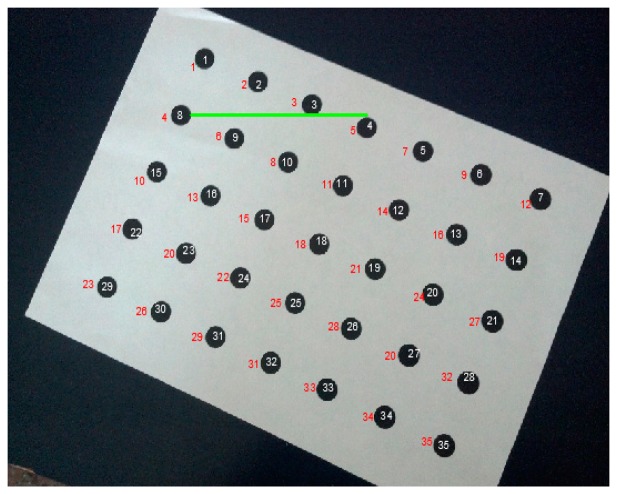
High inclination of a camera's principal axis giving wrong sorting and numbering of control points (red colored points' codes). The point with No. 8 (code in white color inside the circle) is erroneously getting the code No. 4 (red colored code) just because its *j* coordinate on the image (distance from first image row) is smaller than the *j* coordinate of all the other points below the green line.

This last issue severely hinders the success of the calibration adjustment due to possible wrong numbering of the control points on the calibration field. The calibration adjustment function assigns internally to every control point a ground space coordinate according to the distance parallel to the *x* and *y* direction of the columns and rows of the calibration sheet. Therefore for the case of a 5 × 7 (rows × columns) calibration grid and a distance of 37 mm between each row and column, the first point on the top left corner of the calibration field gets ground space coordinates (0, 0, 0), and all the control points on the same (first) row have the same *y* (= 0 mm) and increasing *x* (from 37.0 to 222.0 mm) and the control points in the succeeding columns get *x* coordinates from 0 mm to 222 mm and *y* coordinates from 37 mm to 148 mm. For all the points the height (*z* coordinate) is the same and is 0.0. Therefore the calibration sheet has to be planar in order to achieve the highest accuracy of the calibration parameter calculation. To identify properly a control point at its correct location on the image the software applies a sorting method. For the case of a regular grid of circles of 5 rows and 7 columns the first seven points, identified on the calibration image, are supposed to be visibly closer to the top edge (first image row of the image). However, if the camera's primary axis (implemented by the line connecting the center of lens and the center of the sensor) is rotated very much the software erroneously sorts the control points and the software fails to calibrate the camera (Figure 7).

### 2.4. Software Options

For all the above-mentioned problems and to increase the functionality of a calibration application, we decided to implement a tool with a graphical user interface giving to the user the opportunity to have better control on the calibration algorithms, and therefore to:

- Adjust the threshold algorithm and isolate the circular control points' grid from any noise that might appear on the edges of the calibration images;
- Define the minimum radius of the control point circles since the wide angle lens and the camera position might affect rapidly the variance of the circles' diameter;
- Examine the correct location of the control points on the calibration sheet by providing the correct numbering of all the control points on the image;
- Undistort the still images and create their idealized respective images; the idealized images may be used in single photo photogrammetric procedures to provide rectified photos, *i.e.*, of building facades [20];
- Isolate frames of a video sequence capturing the circles calibration grid and save them as independent images to perform the calibration of the video sensor; since the calibration of the video sensor is performed the undistortion of any video sequence is feasible.

**Figure 8.**
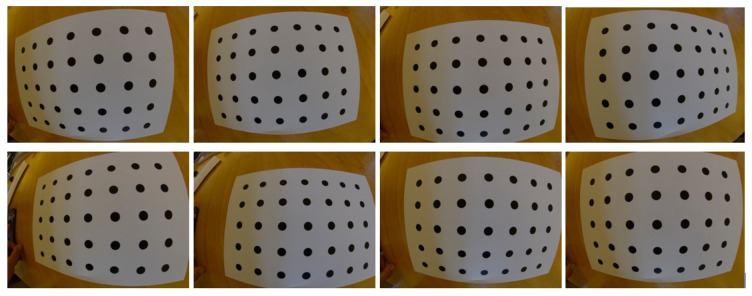
Images captured to perform the calibration.

The created software is characterized by its simplicity and the possibility to interact with other 3D recording software. The user has just to import a set of images using the menus of the Graphical User Interface (GUI) of the software and define the sensor size of the camera to calibrate. The images should be captured in such a way that the calibration page will cover almost the total width and height of the sensor from different locations above of the planar surface of the calibration field (Figure 8). We avoided using a single image calibration setup [21,22], since the result could be inaccurate due to potential asymmetry of the camera construction.

Although the information of the sensor's dimension optionally delivers the calibration parameters in pixels (*f*, *x_o_, y_o_*) it is important for a photogrammetrist to identify the intrinsic parameters of the cameras in millimeters. The rest of the parameters (*k*_1_, *k*_2_, *k*_3_, *p*_1_, *p*_2_) are dimensionless numbers.

The sensor dimensions of any commercial camera is easy to be found using the Digital Photography Review [23]. Reviews of almost all of the new and old commercial cameras, including their basic features, are available on the site. Unfortunately though, there is no appropriate information for the GoPro camera or for any cell phone camera. For the GoPro Hero 3 camera the sensor size information can be derived from the official site of the manufacturing company [24], since it gives the dimensions of a single pixel on the camera image in microns (1.55 μm). Therefore for the highest still image resolution the sensor size is estimated to 6.20 × 4.65 mm. Although, the dimensions of the sensor could be calculated from its diagonal (1/2.3") we decided to use the value calculated by multiplying the image width and height with the pixel size given at the official site of the manufacturing company, because the calculated focal length in the case of the pixel size defined sensor dimension (6.20 × 4.65) was 2.6 mm, a value much closer to the nominal (3 mm) given by the manufacturer of the camera. In the case of the sensor size defined by its diagonal in inches, the focal length was estimated at 2.59 mm. In any case the created undistorted images are not affected at all by the sensor size in mm.

**Figure 9.**
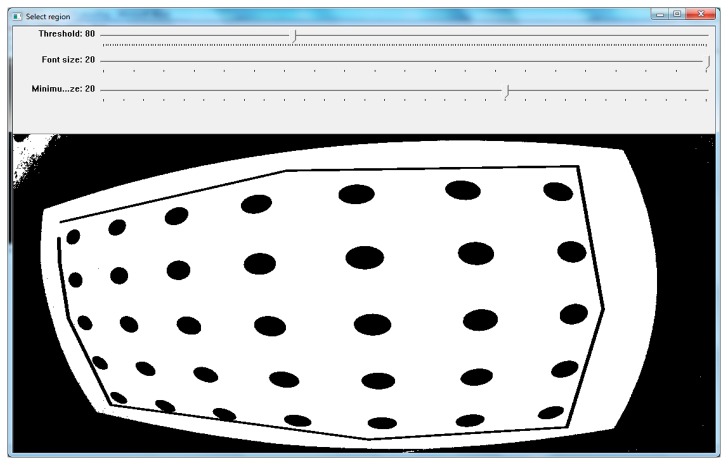
The user defines with the use of the sliders the parameters for the software and defines on the graphical window the region including the control points.

Since the user has added the images of the calibration sheet, has defined the sensor size dimensions and also the distance of the control points grid in mm, the calibration (identification of control points on every image and adjustment to calculate the calibration parameters) can follow. The software browses every image in a graphical window on which the user has to define:

- The threshold value to separate the control points circles from the surrounding area
- The minimum radius of circles to be identified as control points and
- The area including points (Figure 9)

**Figure 10.**
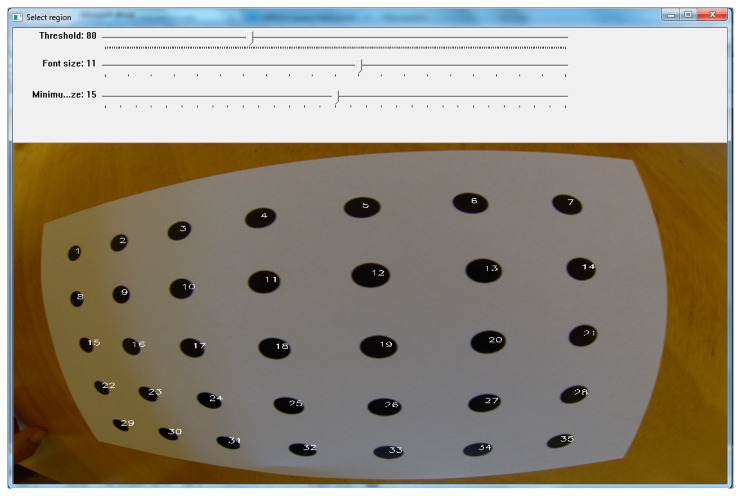
Since the control points are identified correctly the software displays the numbers of control points on the original calibration image.

Although the target identification process is semi-automatic, the total time spent by an average user (namely the students of our post graduate course in IUAV) to adjust the correct parameters and define the area including the targets did not exceed a couple of minutes. Since this operation is performed only once for every camera (or at least once every six months for professional photogrammetric labor) we decided to follow an absolutely correct calibration process, allowing the user to watch and verify the correct location and numbering (Figure 10) of targets on the calibration field, rather than letting the software perform it without any human interaction and operate as a “black-box” device.

**Figure 11.**
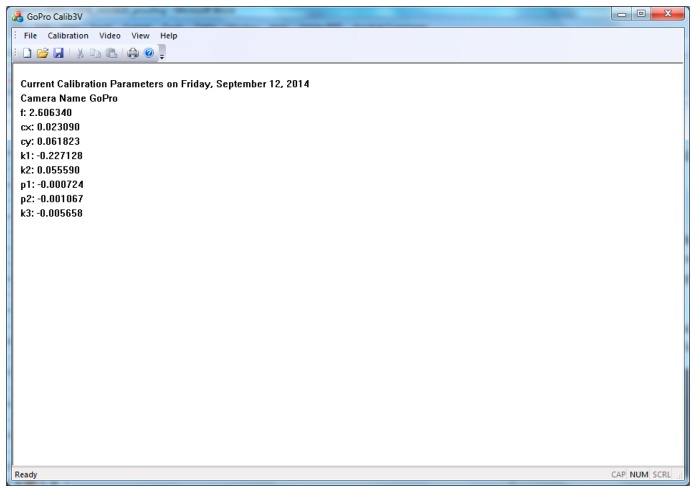
Calibration parameters as they are calculated for the 12 Mpixels still images from the GoPro Hero 3 camera.

After the user has used the images and the control points have been identified, the software prints in the main window application the results (Figure 11), which can be saved for future use in a project (text) file. In our case the focal length of the GoPro camera was about 2.60 mm and the estimated accuracy (Average Reprojection Error) of the calibration was 3.91 pixels. The calibration project file might be used in the future to undistort images taken with the same camera's modes like the one used to capture the calibration images.

**Figure 12.**
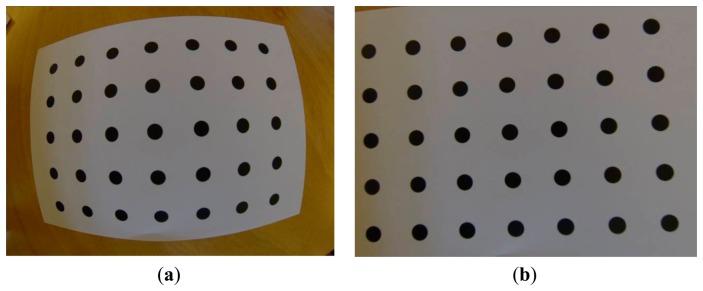
(**a**) Original and (**b**) undistorted images of the calibration field. A small part of the image is lost however considering the geometric correction we can accept the loss.

The image undistortion procedures can follow right after the calibration procedure or can be performed in another occasion since the calibration project file has been stored in the computer's disk file system. The user has just to load the images (at least two) and for every single image, the software performs the undistortioning process (Figure 12a,b).

Finally, although not so much photogrammetry related, an important feature of our software is the possibility to calibrate the sensor when capturing video sequences and perform further the undistortioning of a whole video file.

**Figure 13.**
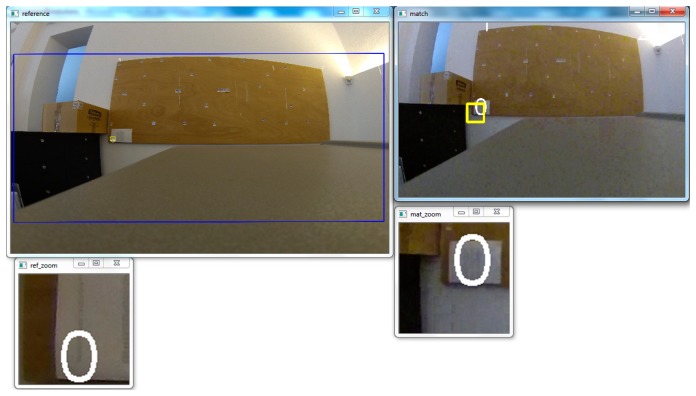
The affine transformation gave the part of the sensor used to capture the low resolution (848 × 480) video frames. The blue rectangle defines the placement of the low resolution image in the full frame image.

Since the part of the sensor that is used to capture every frame of any video mode is smaller than the full resolution of 12 Mpixels we must calculate the exact dimensions of the sensor to perform the calibration. For this reason another simple software applications was created, which is able to calculate the part of the sensor used to create lower resolution (<12 Mpixels) images, using an affine transformation. We can load and browse in two separate graphics windows a full resolution 12 Mpixels image and a smaller one, taken from the same camera location, and if we can identify at least three common points, we can estimate the dimensions of the sensor part used to capture the low resolution (still or video) image (Figure 13), given that the sensor size of the full resolution image is 6.20 × 4.65 mm.

**Figure 14.**
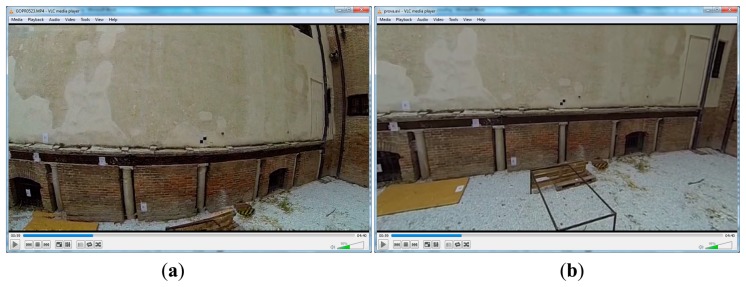
(**a**) Original and (**b**) undistorted video sequences.

The video file depicting the calibration sheet can be loaded in the software. The user can isolate any image from the video and save it as a single calibration image. Following the similar procedure of still images' calibration we can perform the calibration of the video frames. After the completion of the video frame calibration, the user can load an original video file and recreate another one, idealized, with respect to the original, using the undistorted frames (Figure 14).

Finally, the software has the ability to import xml files of camera calibration parameters produced by Agisoft Lens or Agisoft Photoscan. Agisoft Lens is a free software that can calculate the same calibration parameters (*f*, *x*_p_, *y*_p_, *k*_1_, *k*_2_, *k*_3_, *p*_1_, *p*_2_) using a calibration screen, while Agisoft Photoscan is a commercial low cost application that can calculate these parameters using just the conjugate points on overlapping scenes of the recorded objects. Unfortunately, the software does not provide the possibility to create the idealized images, but using the undistortion option of our software we can create the undistorted images produced by any calibrated camera.

## 3. Accuracy Assessment

The use of a widespread and low-cost digital camera combined with easy to use calibration software, such us the free one implemented by the authors (also considering other commercial solutions), offers even non-expert users the possibility to obtain metric data: just considering the immediate results obtainable in teaching or in other different researching areas such as archaeology.

We have tested the use of the GoPro Hero 3 camera on top of a Parrot AR.Drone 2.0 (GPS edition) and have taken a series of images to test the functionality of the camera to provide images of 3D architectural details on a facade not easily reachable from the ground. Both the camera and the drone was controlled from separate mobile devices. The images taken from drone were used to create a complete 3D model of the logia in the university campus giving most accurate results.

**Figure 15.**
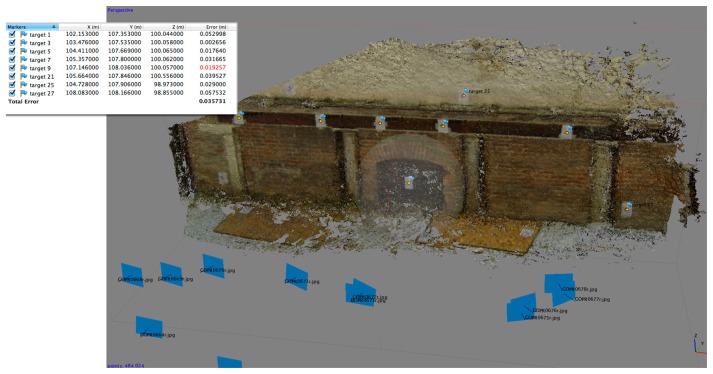
The dense point cloud obtained using undistorted oblique images. RMS error of collected GCPs is about 0.035 m. The test shows that perpendicular undistorted images allow to obtain a higher accuracy.

**Figure 16.**
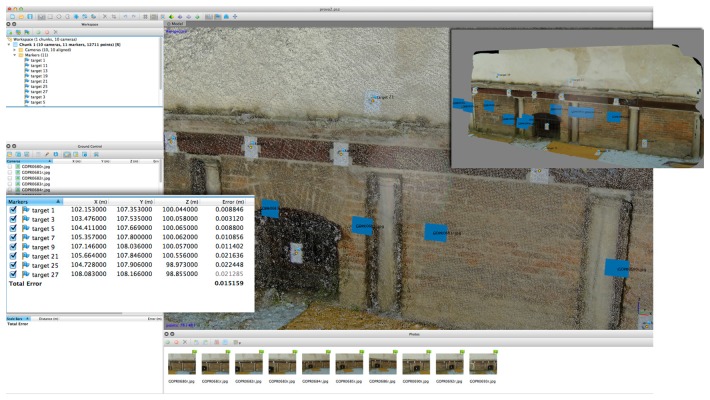
A dense point cloud obtained in Agisoft Photoscan using the GoPro undistorted images. RMS error of collected GCPs is about 0.015 m.

For the 3D photogrammetric processing we have used the software Agisoft Photoscan Professional (version 1.04) and the accuracy in alignment of the GoPro distorted images was about 0.035 m (Figure 15). Using the undistorted ones (Figure 16), the RMS is halved (0.015 m).

Moreover, as shown by some tests we performed using different modes of the camera and capturing schemes and considering other published experiences [21], the geometric scheme that optimizes results, in terms of accuracy, is that of zenithal strip coverage of the objects to be recorded (e.g. parallel to the average plane of the surveyed surface for the architectonical facades), with high overlap (Table 5). Oblique photos demonstrated greater RMS error in the alignment as can also be seen in Table 6 and Figure 16.

This integrated system (GoPro + Parrot AR.Drone) is going to be applied and an in depth test in a archeological surveying campaign that will be carried out in the ancient city of Sepinum, in the south of Italy, to verify its reliability, comparing the results obtained by using a “typical” photogrammetric equipment (such as calibrated camera mounted on a drone).

Within the archaeological documentation or supporting restoration, undistorted images are useful when we want to rectify a planar surface (*i.e.*, building's facade) to further digitize its architectural details. After several tests we have made, we came to the conclusion that the accuracy of the rectification is increased significantly when undistorted images are used.

**Figure 17.**
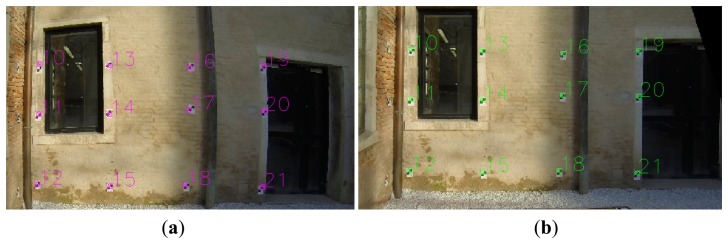
The rectification of the original (**a**) and undistorted GoPro images (**b**) lead to an RMS error of the collected GCPs of 0.0103 m and 0.002 m respectively using the VeCAD-Photogrammetry application [20]. It is also visible the curvature of straight lines on the original image (a).

In the case of the GoPro camera, the originally created images are not appropriate to be used for the rectification of planar facades. RMS error (Table 7), due to the distortion, exceeds 1cm and additionally, points that lie very close to the edges of the image are totally unusable (Figure 17a). The rectification of the undistorted image gave very good results (Table 8) and also the curvature effect (of straight lines in ground space) was totally eliminated (Figure 17b).

**Figure 18.**
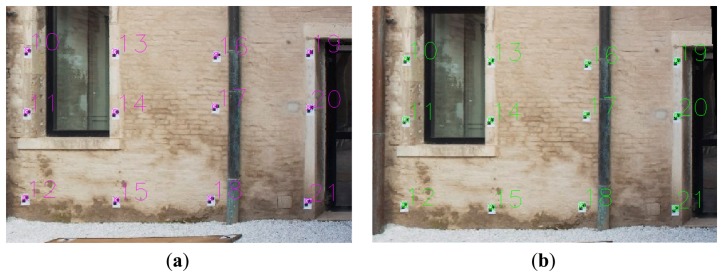
The rectification of the original (**a**) and undistorted NIKON D100 images; (**b**) lead to an RMS error of the collected GCPs of 0.0013 m and 0.0001 m using the VeCAD—Photogrammetry application [20].

The same rectification procedure was used also for original and undistorted images of a dSLR NIKON D100 camera (NIKON CORPORATION, Tokyo, Japan) equipped with a very good quality lens (NIKOR 20 mm/2.8 D AF). The RMS errors (Tables 9 and 10) and rectification residuals (Figure 18a,Tables 9 and 10) and rectification residuals (b) were very small, however they were worse than the undistorted GoPro camera case.

## 4. Conclusions

The possibility to calibrate a low cost camera and especially the GoPro Hero 3 camera which is additionally an action camera is providing lots of benefits:

- The cost of camera is very low and does not exceed 500€;
- The cost of accessories necessary to use it in extreme conditions is zero since it is included in the package;
- The cost of additional equipment to carry the camera for other activities is very small. For UAV applications a low cost drone might be more than enough to carry it and perform remote shootings using just a cell phone applications.

The developed application itself is easy to use by novice users, gives a very good visual corrected result (of the undistorted still images and videos) even to non-photogrammetrists, but provides professional photogrammetrists all the appropriate information to apply the resulting calibration parameters and the derived products for further exploitation in photogrammetric software.

## Figures and Tables

**Table 1.** Camera weight.

**Weight**	**Camera Part**
51 grams	camera
25 grams	battery
76 grams	camera with battery
19 grams	quick-release buckle
106 grams	waterproof housing with quick-release buckle
182 grams	total

**Table 2.** Camera dimensions.

**Dimensions**	**Camera Part**
72 mm × 65 mm × 37 mm	housing size
13 mm × 16 mm	LCD screen size
60 mm × 40 mm × 20 mm	camera size

**Table 3.** Still image resolutions.

**Image Type**	**Width**	**Height**
12 Mp	4000	3000
7 Mp wide	3000	2250
7 Mp medium	3000	2250
5 Mp medium	2560	1920

**Table 4.** Video resolutions.

**Video Type**	**Width**	**Height**	**FPS**	**Image Ratio (Width:Height)**
1080p	1920	1080	29.97	(16:9)
720p	1280	720	59.94	(16:9)
1440p	1920	1440	29.97	(4:3)
4K	3840	2160	14.99	(16:9)
4K Cin	4096	2160	11.99	(17:9)
2.7K	2716	1524	29.97	(16:9)
2.7K Cin	2716	1440	23.98	(17:9)
960p	1280	960	47.95	(4:3)
WVGA	848	480	240.00	(16:9)

**Table 5.** Zenithal undistorted strip errors.

**# Label**	***X***	***Y***	***Z***	***X* Error**	***Y* Error**	***Z* Error**
target 1	102.153	107.353	100.044	−0.006312	0.002943	0.005454
target 21	105.664	107.846	100.556	0.012098	0.008989	−0.015523
target 25	104.728	107.906	98.973	−0.018369	−0.004026	0.012258
target 27	108.083	108.166	98.855	−0.003476	0.002034	0.020901
target 3	103.476	107.535	100.058	−0.001126	−0.000676	−0.002830
target 5	104.411	107.669	100.065	0.001108	−0.005677	−0.006631
target 7	105.357	107.800	100.062	0.006128	−0.000555	−0.008944
target 9	107.146	108.036	100.057	0.009943	−0.003037	−0.004682

**# Total Error**				**0.009183**	**0.004359**	**0.011246**

**Table 6.** Oblique undistorted strip errors.

**# Label**	***X***	***Y***	***Z***	***X* Error**	***Y* Error**	***Z* Error**
target 1	102.153	107.353	100.044	−0.036420	−0.036848	0.011161
target 21	105.664	107.846	100.556	0.027685	0.014427	−0.024244
target 25	104.728	107.906	98.973	0.000180	0.027969	0.007661
target 27	108.083	108.166	98.855	−0.030725	−0.025517	0.041410
target 3	103.476	107.535	100.058	−0.002215	−0.000803	−0.001226
target 5	104.411	107.669	100.065	0.010243	0.011765	−0.008236
target 7	105.357	107.800	100.062	0.021585	0.019010	−0.013242
target 9	107.146	108.036	100.057	0.009631	−0.010095	−0.013273

**# Total Error**				**0.021522**	**0.021218**	**0.019061**

**Table 7.** Ground, image coordinates and their ground residuals of the original GoPro image.

**Point ID**	***X***	***Y***	***j***	***i***	***X* Residual**	***Y* Residual**
1	−1.61	0.829	1720.48	641.73	0.0009	0.0641
2	−0.586	0.809	2195.29	638.97	0.0179	−0.023
3	0.581	0.775	2730.22	683.97	−0.0179	−0.034
4	1.664	0.807	3166.44	723.97	0.0229	0.0249
5	1.663	0.152	3147.07	1006.23	−0.0046	0.0136
6	0.565	0.165	2715.13	959.19	−0.0405	−0.0355
7	−0.592	0.099	2193.27	964.71	0.0054	−0.0356
8	−1.62	0.101	1723.2	965.21	0.0054	0.0132
9	1.641	−0.954	3077.09	1460.36	0.0279	0.0242
10	0.512	−0.924	2656.13	1433.9	−0.0245	0.0064
11	−0.579	−0.943	2190.1	1428.9	0.0092	−0.0046
12	−1.638	−0.916	1731.91	1405.23	−0.002	−0.0137

**RMS Error**	**0.010945**					

**Table 8.** Ground, image coordinates and their ground residuals of the undistorted GoPro image.

**Point ID**	***X***	***Y***	***j***	***i***	***X* residual**	***Y* residual**
1	−1.61	0.829	1703.97	588.51	−0.0013	0.0078
2	−0.586	0.809	2209.41	586.76	0.0015	0.0014
3	0.581	0.775	2815.96	597.28	0.0056	0.0048
4	1.664	0.807	3429.66	561.77	−0.0062	−0.0062
5	−1.62	0.101	1716.3	952.81	0.0022	−0.0052
6	−0.592	0.099	2198.11	953.3	−0.0042	−0.0075
7	0.565	0.165	2768.16	923.41	0.0019	−0.0014
8	1.663	0.152	3342.25	931.63	0.001	0.0013
9	−1.638	−0.916	1730.76	1405.37	0.0059	0
10	−0.579	−0.943	2190.98	1428.61	−0.0078	0.0006
11	0.512	−0.924	2682.77	1432.91	−0.0064	0.0002
12	1.641	−0.954	3209.04	1461.31	0.0079	0.0042

**RMS Error**		**0.002092**				

**Table 9.** Ground, image coordinates and their ground residuals of the original NIKON D100 image.

**Point ID**	***X***	***Y***	***j***	***i***	***X* residual**	***Y* residual**
1	−1.61	0.829	1599.22	569.09	−0.0003	0.0057
2	−0.586	0.809	1876.33	556.1	0.0031	0.0001
3	0.581	0.775	2270.87	544.23	−0.0023	−0.0023
4	1.664	0.807	2735.2	500.86	0.0044	−0.0012
5	−1.62	0.101	1596.36	819.95	−0.0006	0.0007
6	−0.592	0.099	1872.55	826.29	−0.0002	−0.0016
7	0.565	0.165	2259.95	806.93	−0.0056	−0.002
8	1.663	0.152	2725.48	821.06	−0.0017	−0.0005
9	1.641	−0.954	2692.67	1347.2	0.0021	0.0046
10	0.512	−0.924	2228.75	1262.34	−0.0029	0.0004
11	−0.579	−0.943	1870.54	1216.44	0.0051	0.0003
12	−1.638	−0.916	1591.62	1163.88	−0.0012	−0.0041

**RMS Error**		**0.001299**				

**Table 10.** Ground, image coordinates and their ground residuals of the undistorted NIKON D100 image.

**Point ID**	***X***	***Y***	***j***	***i***	***X* residual**	***Y* residual**
1	−1.61	0.829	1599.29	567.49	−0.0013	0.0024
2	−0.586	0.809	1878.83	553.34	0.0011	0.0011
3	0.581	0.775	2281.46	538.24	−0.0018	0.0003
4	1.664	0.807	2767.29	487.13	0.0016	−0.0022
5	−1.62	0.101	1596.37	819.79	−0.0015	0.0004
6	−0.592	0.099	1873.35	825.65	0.0024	−0.0005
7	0.565	0.165	2268.08	804.35	−0.0013	−0.0015
8	1.663	0.152	2752.99	816.5	0.0005	−0.0008
9	1.641	−0.954	2719.06	1354.25	−0.0015	0.0027
10	0.512	−0.924	2236.03	1265.73	−0.0018	0.0013
11	−0.579	−0.943	1871.5	1217.05	0.0057	−0.0002
12	−1.638	−0.916	1591.7	1163.88	−0.0023	−0.003

**RMS Error**	**0.000981**					

## References

[1] Remondino F., El-Hakim S. (2006). Image-based 3D modelling: A review. Photogramm. Rec..

[2] Habib A., Pullivelli A., Mitishita E., Ghanma M., Kim E.M. (2006). Stability analysis of low-cost digital cameras for aerial mapping using different georeferencing techniques. Photogramm. Rec..

[3] Remondino F., Fraser C. Digital Camera Calibration Methods: Considerations and Comparisons.

[4] Fraser C. Automatic Camera Calibration in Close-Range Photogrammetry.

[5] Toschi I., Rivola R., Bertacchini E., Castagnetti C., Dubbini M., Capra A. (2013). Validation test of open source procedures for digital camera calibration and 3D image based modeling. ISPRS Int. Arch. Photogramm. Remote Sens. Spatial Inf. Sci..

[6] Tsai R.Y. (1987). A versatile camera calibration technique for high-accuracy 3D machine vision metrology using off-the-shelf TV cameras and lenses. IEEE Int. J. Robot. Autom..

[7] Zhang Z. (2000). A flexible new technique for camera calibration. IEEE Trans. Pattern Anal. Mach. Intell..

[8] Nakano K., Chikatsu H. (2011). Camera-Variant Calibration and Sensor Modeling for Practical Photogrammetry in Archeological Sites. Remote Sens..

[9] Sirmacek B., Lindenbergh R. (2014). Accuracy assessment of building point clouds automatically generated from iphone images. ISPRS Int. Arch. Photogramm. Remote Sens. Spatial Inf. Sci..

[10] Masiero A., Guarnieri A., Vettore A., Pirotti F. An ISVD-Based Euclidian Structure from Motion for Smartphones.

[11] PhotoModeler Quick Start Guide. http://www.photomodeler.com.

[12] AgiSoft StereoScan. http://www.agisoft.ru/.

[13] MicMac, Software for Automatic Matching in the Geographical Context. http://www.micmac.ign.fr/index.php?id=6.

[14] Schmidt V.E., Rzhanov Y. Measurement of micro-bathymetry with a GOPRO underwater stereo camera pair.

[15] Thoeni K., Giacomini A., Murtagh R., Kniest E. A comparison of multi-view 3D reconstruction of a rock wall using several cameras and a laser scanner.

[16] Wallis Forum 2013. http://www.walis.wa.gov.au/forum/presentations/day-two/slides/3d-modelling-of-underwater-objects-using-photogrammetry-petra-helmholz.

[17] Lehmann Aviation. http://www.lehmannaviation.com/la/la100.php.

[18] Brown D.C. (1971). Close-range camera calibration. Photogramm. Eng..

[19] Fryer J.G., Atkinson K.B. (1996). Camera Calibration. Close Range Photogrammetry and Machine Vision.

[20] Tsioukas V. Simple Tools for Architectural Photogrammetry.

[21] PhotoModeler. http://info.photomodeler.com/blog/using-the-gopro-hero-3-for-`3d-photogrammetry-modeling-and-measuring/.

[22] HTW-Mechlab. http://www.htw-mechlab.de/index.php/intrinsic-camera-parameter-fuer-gopro-hd-hero2/.

[23] Digital Photography Review. www.dpreview.com.

[24] GoPro. http://gopro.com/support/articles/hero3-faqs.

